# *“There’s always somebody that you can identify with”*: a qualitative study of patient perspectives on buprenorphine group medical visits

**DOI:** 10.1186/s13722-025-00540-7

**Published:** 2025-02-05

**Authors:** Mariya Masyukova, Benjamin T. Hayes, Teresa López-Castro, Aaron D. Fox

**Affiliations:** 1 Project Renewal, Inc, 8 East 3rd St, 2nd Floor, Clinic, New York, NY 10003 USA; 2 Albert Einstein College of Medicine, 1300 Morris Park Avenue, Bronx, NY 10461 USA; 3 Montefiore Medical Center, 111 E. 210th Street, Bronx, NY 10467 USA; 4 The City College of New York, 160 Convent Ave, New York, NY 10031 USA

**Keywords:** Buprenorphine, Psychosocial counseling, Group counseling, Group medical visits, Shared medical appointments, Opioid use disorder

## Abstract

**Background:**

Buprenorphine (BUP) treatment has been successfully integrated into primary care and other general medical settings; however, potential BUP prescribers frequently report inadequate training and resources to provide psychosocial counseling as barriers to providing care. Group medical visits, which combine psychosocial support and chronic condition management, have been described for BUP treatment, but few studies have explored what is gained and/or lost by offering BUP treatment in groups instead of individual visits.

**Methods:**

Five focus groups with 3–11 participants each were conducted at an urban community health center that housed a mature office-based BUP treatment program. Participants were persons with opioid use disorder (OUD) who had previously received BUP treatment. A semi-structured interview guide covered the following domains: experience with BUP, experience with group counseling, and preferences for BUP in a group format or individual setting. Qualitative analysis followed a modified grounded theory approach.

**Results:**

Of 33 participants, 28 were male, median age range was 50–54, 20 identified as Hispanic/Latinx, and 24 reported past experiences with substance use disorder treatment groups. Four main themes were: (1) Groups can address the psychological aspects of addiction; (2) Groups introduce positive peer support; (3) Balancing OUD treatment and management of other chronic conditions; (4) Groups must be voluntary.

**Conclusions:**

Findings demonstrate that many persons with OUD taking BUP desire assistance with recovery skills, peer support, and learning about the risks and benefits of BUP. Group medical visits can efficiently deliver these services in primary care, but findings also emphasize that group counseling will be best received when voluntary and group members are committed and authentic.

**Supplementary Information:**

The online version contains supplementary material available at 10.1186/s13722-025-00540-7.

## Background

Buprenorphine (BUP), an effective opioid use disorder (OUD) treatment, has been successfully integrated into primary care and other ambulatory settings [1, 2]. BUP treatment in primary care, which typically includes pharmacotherapy, brief counseling, and routine urine drug testing, reduces non-prescribed opioid use, improves quality of life, and reduces opioid-related overdose risk [3–6]. However, potential BUP prescribers frequently report inadequate training and resources to provide more intensive psychosocial counseling as barriers to prescribing [7, 8]. Many patients receiving BUP in primary care also desire higher levels of psychosocial support than can be provided in focused medical encounters [9, 10]. 

The standard of care for OUD treatment in the United States includes combined pharmacotherapy and psychosocial counseling with opioid treatment programs offering both services. Some evidence suggests that patients receiving methadone treatment and counseling may reduce opioid misuse more than those only receiving pharmacotherapy [11]. However, in several randomized controlled trials, adding intensive psychosocial counseling to office-based buprenorphine treatment has not improved average treatment outcomes in study samples [12–15]. Clinical trials have added cognitive behavioral therapy, contingency management, and enhanced drug counseling to office-based BUP treatment; however, participants receiving standard care, meaning pharmacotherapy and brief physician visits for medical management, have had similar treatment retention and non-prescribed opioid use in comparison to standard BUP plus intensive psychosocial services [16]. Nonetheless, in observational studies, BUP patients who attend more psychosocial services tend to have better outcomes than patients who do not; [17, 18] therefore, making services available may benefit some patients. The ideal approach to providing patient-centered BUP treatment with optimal psychosocial support is still unclear.

Group Medical Visits are a model for multidisciplinary patient-centered care that combines psychosocial support and chronic condition management. There has been growing interest in BUP group medical visits in the Veterans’ Affairs health system and other community providers [19–23]. The general approach to group medical visits has been described in detail elsewhere [21, 24–26]. One categorization proposes two group-based BUP models: “group psychotherapy” provided by a behavioral health specialist coupled with asynchronous BUP prescribing by a medical provider or “shared medical appointments” wherein BUP prescribing and group counseling occur concurrently either by a single medical provider or multidisciplinary team of clinicians [23]. Common elements of group-based BUP are a multidisciplinary clinical team (usually a BUP prescriber and a behavioral health specialist), peer support, instruction on self-management skills, and either addiction-related education or evidence-based psychotherapy [23]. Most described group-based BUP models have not incorporated preventive medical care or management of other co-morbid chronic conditions. When used to manage other chronic medical conditions, like diabetes, group medical visits are associated with improved adherence, symptom reduction, decreased health care utilization, improved access to care, and increased self-efficacy when compared with usual care [27–29]. Group medical visits for BUP could be an efficient way of adding additional psychosocial support to BUP.

Though group medical visits appear promising for OUD and have been widely used for other chronic conditions, BUP group medical visits have not been rigorously studied, and patient perspectives on group-based BUP models have only been described in one prior study [30]. Exploring potential psychosocial supports to accompany BUP could also improve care for patients who experience office-based BUP as inadequate [10]. This study’s objective was to describe patient perspectives on what could be gained and/or lost by offering BUP as a group medical visit instead of an individual encounter.

## Methods

This qualitative study followed the Consolidated Criteria for Reporting Qualitative research checklist [31]. It was performed from an interpretivist perspective: we sought to understand participants’ attitudes based in their reported experiences and socio-ecologic context rather than assuming a single “truth” regarding the value of group-based BUP treatment. Interpretivism views reality as constructed by the mind of individuals, and therefore the researcher seeks to uncover deeper meaning by asking subjects to reflect upon their experiences and understanding [32]. Our research team included two male general internists, one female senior medical student with training in qualitative research methods, and a clinical psychologist with addiction research training. Two investigators (MM & ADF) had experience initiating a pilot BUP group medical visit program for patients with OUD. This study was approved by the Institutional Review Board of Albert Einstein College of Medicine/Montefiore Medical Center.

### Setting

Focus groups took place at a Federally-Qualified Health Center (FQHC) in the Bronx, NY, where approximately a dozen general internists provided BUP within routine primary care. The BUP program has been described in detail elsewhere [2]. The FQHC serves a low-income urban neighborhood that is 57% Hispanic and 39% non-Hispanic black. Over 65% of patients have public insurance. Social workers were available at the health center, but neither intensive psychosocial counseling nor group-based treatment was routinely included with BUP treatment [2]. 

### Participants

A convenience sample of people with OUD was recruited via advertisements in the health center, physician referral, and direct contact from a list of BUP patients who had given consent for researchers to contact them about BUP studies. Inclusions were self-reported OUD and prior office-based BUP treatment. We did not require prior experience with any specific type of group substance use disorder (SUD) treatment, because we wanted to include participants with a diversity of perspectives toward groups.

### Procedures

Investigators facilitated five focus groups with 3–11 participants (median 6 participants) in August 2014. Before conducting each focus group, investigators described to all participants present the objectives of the study, including the risks of participation (i.e., discomfort discussing sensitive information in a group setting and potential breach of confidentiality). All participants provided written informed consent. One focus group with 3 participants was composed of patients who participated in a BUP group medical visit program piloted by the investigators, while other focus groups were composed of patients without a clinical relationship to investigators who had group treatment experience in other settings or no group treatment experience. Focus groups were held in a private conference room. One researcher acted as facilitator (MM) and another observed and took notes (ADF). Sessions were about one hour in length and were audio-recorded. A cash incentive, two-way public transit pass, and refreshments were provided to each participant. The cash incentive was $10 for the first focus group, and these participants gave feedback that it was too little, so subsequent focus groups offered a $20 incentive. Participants completed demographic surveys prior to the focus group. No unique identifying information was linked to each participant’s qualitative data (e.g., age was collected in categories of 5 years; data was pooled to describe the sample in aggregate). There were no repeat interviews.

A semi-structured interview guide was designed for this study to capture patients’ past experiences with groups in SUD treatment and perspectives on incorporating groups into BUP treatment in primary care. The biomedical literature was reviewed to confirm that other studies had not addressed these questions. Investigators then developed four questions and sought feedback from a qualitative research affinity group in Montefiore’s Division of General Internal Medicine. This group included BUP clinicians and experienced qualitative researchers who confirmed the face validity of questions and made recommendations on wording to enhance the clarity. The interview guide covered the following domains: experience with BUP, experience with group counseling, and preferences for BUP in a group or individual format (see Appendix). The facilitator framed each question in multiple ways and probed for elaboration when applicable.

Audio recordings were transcribed manually by one researcher (MM). Data integrity was verified by cross-checking transcripts. Transcripts were coded individually by two researchers (MM and ADF) and analyzed using a modified grounded theory approach, as described by Auerbach and Silverstein [33]. After reading and coding each transcript, researchers met to discuss the coding scheme and agree upon final codes. Researchers listed “repeating ideas” and described the theoretical constructs that emerged from this iterative process. Data were organized into categories of increasing complexity, starting from relevant text categorized into “repeating ideas,” organized into “themes,” and then expanded into a “theoretical narrative” relevant to the initial research question. Focus groups were conducted until theoretical saturation was reached (i.e., when no additional “repeating ideas” were detected in the source text during analysis of the fifth focus group).

## Results

### Participant characteristics

Of 33 participants, 28 were male, median age range was 50–54, 20 identified as Hispanic/Latinx, eight identified as Black (see Table 1). Twenty-four reported past experiences with group SUD treatment (most in settings outside of the FQHC and not specific to BUP treatment). No participants dropped out of the study.

**Table 1:** Demographic and Clinical Characteristics for Focus Group Participants (*N* = 33)

Characteristic	*N* (%)
Age Range (Median)	50–54
Male	28 (85)
**Race/Ethnicity**	
Hispanic/Latinx	20 (61)
Black	8 (24)
Other	3 (9)
Declined/missing	2 (6)
**Previous experience with group SUD Treatment (any)**	24 (73)
*Both* staff-led and participant-led SUD groups	18 (55)
Participant-led groups only	3 (9)
Staff-led groups only	3 (9)
No prior experiences with SUD groups	3 (9)
Declined/missing	9 (27)
**SUD = substance use disorder**	

Participants discussed their experiences with BUP in primary care and other group-based OUD treatment, highlighting four main themes relating to what could be gained or lost with group visits: (1) Groups can address the psychological aspects of addiction; (2) Groups introduce positive peer support; (3) Balancing OUD treatment and management of other chronic conditions; (4) Groups must be voluntary. More details regarding these themes follows with quotes from participants that represent prevailing attitudes.

### Groups can address the psychological aspects of addiction

Participants frequently expressed the belief that OUD had distinct psychological or “mental” components and physical components. Though participants generally believed that BUP was essential to address physical symptoms (e.g., preventing withdrawal), most also expressed that they struggled with the psychological dimensions of addiction, such as craving, regulating emotions, and maintaining motivation to continue treatment. Group counseling was generally viewed as a favorable addition to traditional BUP treatment in that it offered a therapeutic context to cultivate recovery skills:

*“[BUP] helped physically. But I still had the mental addiction. Wanted to use. And that’s one of the areas from the [BUP] that I’m still having trouble with*,* because it’s not stopping the cravings… It’s stopping the cravings physically*,* but mentally*,* it’s still there…*.

Not all participants believed that groups alone carried this benefit. Many had a trusted BUP practitioner who both wrote prescriptions and provided counseling and support that address the psychological aspects of addiction. The duration of therapeutic relationship, personal attributes of practitioners, and breadth of skills in addressing “non-medical” issues contributed to strong therapeutic alliance that some participants had with their BUP prescriber. One participant described this bond:

*“I’m good with my doctor, you know. I could talk to her about anything…that I’m going through. And she understands. She’s not only my doctor, she’s a friend, she’s also a counselor as well, all in one. So I’m grateful.”*

BUP’s pharmacologic effects seemed to support participation in and responsiveness to counseling. Participants felt more capable to engage with their thoughts and emotions when taking BUP, which would facilitate productive participation in groups. The following participant felt strongly that BUP allowed them to explore the psychological dimensions of OUD treatment:

*“I’m more… aware. I’m more awake. I’m more functional. I can think better. So as far as the [BUP] is concerned, when I’m in groups, I can actually speak what I’m really feeling, because I’m more on point, and I’m more, again, functional.”*

Generally, participants valued treatment with multiple modalities, framing counseling or “groups” as a complementary component of BUP. They emphasized that, ideally, treatment modalities would be coordinated with multiple providers working together to help patients reach their treatment goals. One participant enthusiastically described team-based care:

*“With my doctor, there’s another counselor there. And he asks me how I’m doing in the week, do I have any problems…he works side to side, when I go to see the doctor for my [BUP], I’m still getting counseling. So it’s a little bit of everything.”*

Not all participants who valued groups specifically desired counseling focused on behavioral change or emotion regulation. Many participants expressed an unmet need for education about BUP pharmacology and interactions with other medications and comorbidities. One participant specifically described their interest in learning about pharmacotherapy:

*“I think a good group would be just to talk about risks and side effects. Cause I don’t really talk too much about that with the doctor…I was good, I wasn’t on methadone anymore, certain milligrams they talked about, but we need to talk more about side effects and drinking.”*

### Groups introduce positive peer support

Many participants’ OUD treatment goals included maintaining or expanding a supportive social network. Multiple participants felt that connecting with peers who did not use drugs, meaning other patients in groups not peer recovery specialists, contributed to their treatment success. Peers who were actively using or social situations where drug use occurred could lead to potential setbacks. Participants expressed desires to be in supportive settings and wanted to minimize exposures that could overwhelm their present ability to manage cravings or triggers. The following participant explained the influence of “negative” networks:

*“If you surround yourself with positive people, you won’t be there. But if you’re around the same people you were when you were getting high, you’re going to get high. You’re going to be around negative people, that’s what it is, negativity.”*

Participants in multiple focus groups expressed interest in building community with others being prescribed BUP. Isolation was perceived as a challenge; one participant summarized the desire to identify others who took BUP but having difficulty doing so:

*“I already feel like a minority because I’m on [buprenorphine] among prior opiate users*,* cause most of the people I know are in the [methadone] clinics… and I can count on one hand the amount of people I know that made the jump on [buprenorphine]*,* got off the methadone. I guess what I’m saying is that I don’t really know anyone else on [buprenorphine] and I’ve been on it for 7 years*,* here.”*

In addition to reducing isolation, the group setting was seen as an opportunity to share information and resources with peers. This was modeled in one focus group by a participant sharing harm reduction agency contact information with another research participant:

*“Different people have different information. And it’s not even the counseling. We get it all from each other. Like I pulled my card out*,* they may not have known about that place. That’s information that I’m giving to them.”*

Focus group participants attributed groups’ benefits to the authenticity of group participation, sincerity of interactions, and subsequent validation of shared experiences. The group setting was a desirable opportunity to learn and practice psychosocial skills alongside peers who were exposed to similar challenges. Some participants felt that their peers were an unparalleled resource because of shared lived experiences of drug use and recovery:

*“The feedback that we get from ourselves is gonna be better than what we get from the doctor. Cause we know more about each other, can help each other better than anyone else can.”*

Participants also highlighted their peers’ capacity to provide empathy and validation:

*“The feedback that we get from ourselves is gonna be better than what we get from the doctor. Cause we know more about each other, can help each other better than anyone else can.”Being in groups with people that I know were going through the same thing that I was going through just validates it even more that I’m doing the right thing and the [BUP] is working.”*

Though group peer interactions were mostly viewed positively, a minority of participants expressed concern about confidentiality in group settings. Perceived risks of breaches in confidentiality were framed as a potential barrier to fully disclosing thoughts and feelings.

*“I can feel that in a group setting, they can’t really express their real feelings, because then people take it outside, they see him on the street, they say oh look, he was this and that, but what’s said in the group is supposed to stay in that group, and it doesn’t always work that way. People don’t express themselves as much in a group setting as they would individually with a doctor.”*

### Balancing OUD treatment and management of other chronic conditions

Some participants expressed concerns that with group-based BUP, their other chronic medical conditions may receive less attention than OUD. For these participants, seeing a primary care practitioner was preferable, because OUD and other conditions could be efficiently managed concomitantly.

Participants felt that combining their other medical needs and BUP within primary care was not only convenient, but also led to better medication safety and continuity of care. One participant in favor of one-on-one care explained:

*“My first doctor in the [BUP] program, I liked the relationship we had, and I liked the fact that he could take care of my other needs. My high blood pressure, and my others, so I could come to one place and have all my needs taken care of, not just [BUP]. And he made it so that I only had to come here once a month and I got everything taken care of.”*

However, others noted that a problematic over- or under-emphasis on OUD could also occur in individual-based BUP. Some participants felt that their provider paid disproportionate attention to BUP at the expense of management of other conditions; others were disappointed in their provider for focusing on their medical conditions without enough regard to their OUD. One participant who desired more time spent on medical management explained:

*“Sometimes I feel as if*,* because my doctor is my primary care physician*,* as if the [BUP] is like the tail wagging the dog…I have terminal illness. And it’s untreatable…in the whole scheme of things in my life… it just sometimes seems like the time spent on [BUP] is just kind of out of proportion with the way everything shakes down.”*

Another participant desired to spend more time on discussing OUD with their physician, *“I feel more comfortable when I go to my counselor in the program*,* not with my doctor…because when I go to see my doctor*,* she only focuses on my problem with the HIV.”*

Nonetheless, some participants expressed that group settings could also benefit management of medical comorbidities due to the potential to share knowledge and peer experience:

*“If you’re not the only person going through that experience*,* wouldn’t the group setting then be better*,* because…he could tell you something…you don’t have to take another medication*,* let’s try to wipe down the medications*,* try not to overdose.”*

Overall, opinions about management of medical comorbidities did not seem to affect preference for individual- or group-based treatment or acceptability of BUP treatment in general.

### Groups should be voluntary

Participants reflected a range of perspectives on the necessity and added value of group counseling in OUD care. While some believed that groups were essential to their own successful recovery, most participants felt that pharmacotherapy and brief medical visits would be adequate for some individuals, and that group participation should be a choice left up to the individual. One participant noted how he greatly appreciated the choice to tailor treatment to his needs and preferences, which in this case entailed solely pharmacotherapy and medical visits:

*“I feel fortunate to say that I wasn’t doing any groups when I was on [BUP] and I didn’t need the group. I was just focusing on what I want to achieve.”*

The following person summarized a common theme expressed multiple focus groups -- that group counseling is not universally effective or preferred: *“Some people take to group settings*,* some people don’t.”*

Voluntary participation was felt to foster a successful group environment. Participants believed that with group membership as a choice rather than a requirement for BUP, group members would be more motivated and genuine. Participants consistently emphasized the importance of authenticity among group members, with the greatest potential for trust and group cohesion with group participants who were respectful and striving for positive changes. This participant explained a negative experience with a group member:

*“You’ve got some groups where guys just go there just to BS…it makes me just want to get up and walk away. And you got groups where you can be able to share, you get to open up.”*

Participants also commonly highlighted the importance of voluntary, convenient, efficient, and accessible treatment options. These were universally appreciated and regarded as significant contributors to treatment engagement and benefit. One participant who preferred the option of one-on-one buprenorphine treatment was concerned that mandating groups might be overly time consuming:

*“I like the way it is now. Because each time, it’s like you’re in and out. You’re not in the spot where you’re waiting for the next man…just in and you’re out…. You see a doctor, get your prescription, you’re done.”*

## Discussion

Our study of BUP patients’ attitudes toward OUD treatment groups provides insights on their desired types of psychosocial support and the potential role for BUP group medical visits. Participants wanted psychosocial counseling and opportunities to expand positive social networks, but many were also satisfied with individual models of BUP treatment. Participants stressed that group participation should be voluntary, and that group attendance should not be required to receive BUP prescriptions. Taken together, the identified themes underline the importance of low-barrier, non-coercive OUD care and the value of authentic peer support– insights that may inform BUP program administrators and/or clinicians looking to improve their models of care delivery.

Prior research has demonstrated that BUP group medical visits are feasible and valued by participants [20, 30], and our study adds knowledge on what could be gained and lost when BUP treatment is primarily delivered in group encounters. In particular, participants were concerned about management of medical comorbidities, which was not identified as a concern in prior research on BUP group medical visits [24, 30]. When integrated into primary care, BUP can facilitate improved management of non-OUD chronic conditions, including HIV infection [34]. It would be possible to deliver some medical care during a group medical visit, such as influenza vaccines or smoking cessation interventions, which could improve efficiency; however, if groups are organized around management of OUD alone, individualized chronic disease care could be compromised. Prospective clinical trials should rigorously evaluate the group medical visit model with attention to OUD outcomes, but also whether medical conditions can be managed sufficiently in comparison to standard BUP treatment in primary care.

Consistent with literature on the therapeutic mechanisms of group approaches [35, 36], our study participants identified multiple ways in which group participation could benefit them. Like another qualitative study with patients from a BUP group medical visit program, participants spoke about benefits of shared experience, validation, education, feedback, and cohesion [30]. Many found groups to be desirable, and, for some, even indispensable to successful BUP treatment. Because most participants had prior experience with group SUD treatment, many insights were substantiated by their lived experience. The variation in participants’ views highlighted that possible benefits of individual vs. group treatment models may affect patients differently based on their personal preferences and needs. Thus, there cannot be a “one size fits all” approach to groups. Our findings underscore that diverse group elements should ideally be available to those seeking OUD treatment; patients’ context, clinical needs, and expressed preferences could determine engagement with psychoeducation, skill-building, peer support, interpersonal processing, and other group elements and processes [37]. Participants also emphasized that intentional group composition (e.g. connecting peers with similar treatment goals) may be conducive to trust and cohesion, if it does not compromise voluntariness of group participation.

The way that participants viewed OUD as having physical, psychological, and social components is highly consistent with a biopsychosocial framework of SUD treatment [38]. Participants noted how physical symptoms existed alongside psychological experiences, like cravings and motivations, and within social interactions like peer pressure and support. Controlling craving and withdrawal allowed participants to engage effectively with counseling and skill building. The biopsychosocial paradigm acknowledges the interplay of biological, psychological, and social factors in the origin and maintenance of SUDs and argues that optimal care involves tailoring treatment to the unique, multifaceted needs of each patient. For some, as participants highlighted, this may mean strong social reinforcement and modeling for recovery in addition to pharmacotherapy; for others, directed psychotherapy may help address comorbid mental health conditions. Therefore, group medical visits could efficiently provide multiple aspects of needed care, but they are also only part of a spectrum of services and supports desired by people with OUD.

This qualitative study has limitations in its execution and scope. Focus groups were conducted at single urban FQHC and may not represent the views of persons with OUD in other geographic areas or clinical settings. Focus group participants were mostly male middle-aged persons of color, and other groups, such as women or nonbinary people and young adults, may have different preferences and OUD treatment needs. Some focus groups participants, specifically the participants who were part of a prior group medical visit pilot, had a clinical relationship with some of the investigators, which may have influenced their comments during focus groups. The study focused on group medical visits as they apply to BUP treatment, but important topics of confidentiality, stigma, and motivations for OUD treatment were not discussed in detail. Future research is critical in these areas—SUD treatment remains highly stigmatized in the U.S. and patients’ experiences will be crucial in finding solutions. Participants also had variable experience with prior group treatment, which may have influenced how they understood what a BUP group medical visit would entail. We did not explore other potential BUP treatment models that could be preferable to participants (e.g., telehealth). Other topics that may affect attitudes toward OUD treatment in this population, such as experiences with structural racism, prior methadone treatment, or presence of other comorbid SUDs were also not be explored in these focus groups.

With two decades of clinical experience and numerous clinical trials demonstrating efficacy, BUP treatment in primary care will continue to be a first-line OUD treatment; however, there is room for practice improvement [5, 39]. Our findings demonstrate that many persons with OUD taking BUP also desire assistance with recovery skills, peer support, and learning more about the risks and benefits of treatment. Group medical visits may be one way to efficiently deliver these services in primary care, but our findings also emphasize that group counseling will be best received when voluntary and group members are committed and authentic. While prioritizing patient preference in developing and implementing new models for BUP treatment, it appears that group medical visits could fill unmet patient needs.

## Electronic supplementary material

Below is the link to the electronic supplementary material.

Supplementary material 1

## Data Availability

No datasets were generated or analysed during the current study.
