# Supplementary material for: “There’s always somebody that you can identify with”: a qualitative study of patient perspectives on buprenorphine group medical visits
Source: Addict Sci Clin Pract. 2025 Feb 5;20:8. doi: 10.1186/s13722-025-00540-7 (PMC11796086; doi:10.1186/s13722-025-00540-7)
Supplement: Supplementary file 1 — Supplementary material 1 [file 13722_2025_540_MOESM1_ESM.docx]

Appendix:

(1) What has been your experience getting buprenorphine (Suboxone) treatment through your primary care doctor? (2) What has it been like for you to participate in “groups” for substance use treatment? (3) Which option from the following two would be better for you and why? Option 1 is receiving buprenorphine treatment from your primary care doctor, seeing them alone in the office for a shorter visit, and Option 2 is a group of people who are all receiving buprenorphine treatment that meets with the doctor at the same time for a longer visit. (4) What do you think about Option 2?
